# Inhibition of the negative effect of high glucose on osteogenic differentiation of bone marrow stromal cells by silicon ions from calcium silicate bioceramics

**DOI:** 10.1093/rb/rbz030

**Published:** 2019-09-30

**Authors:** Xixi Dong, Xiaoya Wang, Min Xing, Cancan Zhao, Bin Guo, Junkai Cao, Jiang Chang

**Affiliations:** 1 Stomatology Department, General Hospital of Chinese PLA, 28 Fu Xing Road, Beijing 100853, PR China; 2 State Key Laboratory of High Performance Ceramics and Superfine Microstructure, Shanghai Institute of Ceramics, Chinese Academy of Sciences, 1295 Dingxi Road, Shanghai 200050, PR China

**Keywords:** high glucose, silicate, osteogenic differentiation, bone tissue engineering

## Abstract

Human bone marrow stem cells (hBMSCs) are exploited for miscellaneous applications in bone tissue engineering where they are mainly used as seed cells. However, high glucose (HG) environment has negative impacts on the proliferation and osteogenic differentiation of hBMSCs, thus reducing the bone formation in diabetic patients. In our former research works, we discovered that silicon (Si) ions extracted from silicate-based bioceramics are able to stimulate the proliferation and osteogenic differentiation of hBMSCs under normal culture condition. This study aimed to investigate if Si ions could prevent HG-induced inhibition of proliferation and osteogenesis of hBMSCs. We found that 2.59 ppm concentration of Si ions promoted the proliferation of hBMSCs under HG condition. The results from alkaline phosphatase (ALP) activity assay, Alizarin red S staining and quantitative real-time PCR analysis of osteogenic genes (BMP2, RUNX2, ALP, COL1 and OCN) demonstrated that the 15.92 ppm concentration of Si ions prevented HG-induced inhibition of the osteogenic differentiation of hBMSCs. Moreover, application of Si ions reduced the level of reactive oxygen species in HG-treated hBMSCs. In HG-treated hBMSCs following 15.92 ppm Si ions treatment, activation of BMP2/SMAD signaling pathway was detected, as indicated by the increased expression of BMP2 receptors and its downstream genes such as *SMAD1*, *SMAD4* and *SMAD5*. Taken together, we provide evidence that the specific concentration of Si ions compensated HG-induced inhibition of proliferation and osteogenic differentiation of hBMSCs through antioxidant effect and modulation of BMP2/SMAD pathway. The results suggest that silicate-based bioceramics might be good scaffold biomaterials for bone engineering applications in diabetes patients.

## Introduction

Clinical osseous insufficiency is described as a gap in bone integrity and is initiated by factors such as inherited defect, pain or operating resection [1]. The advances in bone tissue engineering have boosted the development of alternative approaches for the repair and reconstruction of bone defects. Seed cells and scaffolds are considered as the two important factors in bone tissue engineering. However, human bone marrow stem cells (hBMSCs), one of the most important seed cells with multiple differentiation ability [2], has enormous challenges in bone tissue engineering under high glucose (HG) condition. 

Growing number of studies indicate that hyperglycemia impairs bone quality, though the underlying mechanisms remain unclear. *In vitro* experiment has proven that HG microenvironment exerts a consequential inhibitory action on the osteogenic differentiation and growth of hBMSCs [3, 4] and MC3T3-E1 cells [5], indicating that the negative effect of HG condition on hBMSCs might be closely related to the diabetes associated decrease of bone regeneration ability. Previous publications conveyed that HG hinders the osteogenic differentiation of BMSCs via modulating the BMP signaling pathway which downregulates the levels of osteogenic markers such as osteocalcin, alkaline phosphatase (ALP) and Runx2 [6]. In addition, it was demonstrated that, under HG condition, Runx2 induces matrix mineralization by upregulating the expression levels of osteogenic markers [7]. Furthermore, premature aging, genomic instability and telomere changes were detected in HG-treated BMSCs, which led to decreased rate of BMSCs proliferation [8].

Silicate bioceramics have been found to stimulate bone regeneration and are considered as promising scaffold materials for bone tissue engineering. Our previous studies have shown that silicate bioceramics had good biocompatibility, safety and osteoconductivity [9, 10], and Si ions released from silicate-based bioceramics pointedly enhanced the osteogenesis and growth of BMSCs, and stimulated angiogenesis of endothelial cells [11–14]. Our recent study has demonstrated that the Si ions in the concentrations of 2.59 and 15.92 ppm stimulated the proliferation and osteogenic differentiation of hBMSCs, respectively [15]. And it is reported that 30 mM glucose can inhibit the proliferation and differentiation of BMSCs [4]. Therefore, our hypothesis is that Si ions from silicate-based bioceramics may be also effective in compensating the adverse impact of high levels of glucose on the osteogenesis of hBMSCs.

This study aimed to investigate whether the Si ions extracted from calcium silicate (CS) could alleviate or compensate the inhibitory action of high levels of glucose on the proliferation and osteogenesis of hBMSCs. The possible mechanisms related to the effect of Si ions on hBMSCs under HG condition, in particular the effect on osteogenic signaling pathways such as the BMP2 signaling pathway was also investigated.

## Materials and methods

### Preparation of biomaterial extracts

CS powders were obtained as previously described chemical co-precipitation approach [16]. Briefly, the CS powder was soaked in 200 mg/ml of basal medium for human mesenchymal stem cells (Cyagen Biosciences, USA). Following the incubation at 37°C for 24 h in an incubator containing 5% CO_2_, the mixture was centrifuged at 2000 *g* at room temperature for 10 min. Then, the supernatant was collected and filtered through a microfilter (Millipore, 0.22 mm) before storage at 4°C according to the ISO10993-1 standard [17]. The content of Si ions in the extracts was determined by inductively coupled plasma optical emission spectroscopy (710-ES, Varian, USA) [15].

### Cell culture

The hBMSCs were purchased from Cyagen Biosciences Inc. (USA) and seeded using the basal medium for human mesenchymal stem cells (Cyagen Biosciences, USA) added with glutamine (Cyagen Biosciences, USA), 10% hBMSCs-specific FBS (Cyagen Biosciences, USA), 100 mg/ml streptomycin and 100 U/ml penicillin (Cyagen Biosciences, USA). The culture protocol was performed in an incubator following the method described in our previous work [18]. Since our previous study showed that 2.59 and 15.92 ppm optimal concentrations of Si ions in the medium, respectively, enhance the proliferation and osteogenic differentiation of hBMSCs [15], we selected the Si ions concentrations of 2.59 and 15.92 ppm for cells proliferation and osteogenic differentiation experiments, separately. 

### Cell proliferation assay

The proliferation assay was performed to determine the effect of Si ions on proliferation of hBMSCs under HG condition. The hBMSCs (1 × 10^3^ cells/well) were seeded in 96-well plates and cultured in a humidified 5% CO_2_ incubator at 37°C. Cells were cultured in different medium: normal glucose (NG) 5.5 mM, 5.5 mM + 2.59 ppm (NG + 2.59), high glucose (HG) 30 mM and 30 mM + 2.59 ppm (HG + 2.59). After culturing for 1, 3 and 7 days, the Cell Counting Kit (CCK-8) assay kit (Beyotime) was applied to detect the viability of hBMSCs following the manufacturer recommendations. Briefly, at each time point, the culture medium was replaced by the fresh medium added with the CCK-8 reagent (10:1) and the cells further incubated in the same culture condition for additional 2 h. Finally, the ELX Ultra Microplate Reader (Bio-tek, USA) was used to spectrophotometrically measure the optical density (OD) at 450 nm. The results were expressed as units of OD absorbance value.

### ALP assay

ALP staining was performed 10 days after culture in 24-well at a density of 1 × 10^5^ cells/well according to a previously protocol [19]. Briefly, after washing with 1 × PBS, cells were fixed for 15 min in 4% paraformaldehyde at room temperature. Next, the cells were washed using the deionized water and subsequently incubated in 166 ml/4 ml naphthol/fast blue solution (Sigma) in the dark for 20 min at 37°C following the manufacturer’s protocols. The stained cells were subsequently examined using the Leica microscope coupled with a digital camera (DMI 3000) and photographed.

The ALP Assay Kit (Colorimetric, Abcam, UK) was also used to measure ALP activity. In brief, cell lysates were harvested and after centrifugation (10 000 rpm) for 10 min at 4°C, the Pierces BCA Protein Assay Kit (Thermo ScientificTM, USA) was employed to determine the total protein concentration [18]. Next, the supernatant was aliquoted (100 μl) and combined with 200 μl *p*-nitrophenyl-phosphate solution (pNPP: Sigma, St. Louis, USA) in a 96-well plate. Finally, the Microplate Reader (Bio-tek, USA) was used to measure the absorbance at 405 nm which was used to calculate ALP activity. The concentration of cell total protein was used for normalization.

### Alizarin red S staining

Cells (1 × 10^5^ cells/well) were cultured in triplicate in diverse media for 21 days in 24-well plates. Culture media were added with b-glycerophosphate (10 mM), dexamethasone (100 nM) and ascorbic acid (0.05 mg/ml). Alizarin red S staining was carried out as described in a previous paper [20]. Briefly, after rinsing twice with PBS, cells were fixed for 10 min in 10% formalin at room temperature. Subsequently, the cells were washed several times using distilled H_2_O and stained by 2% (w/v) Alizarin red (Sigma) for 5 min at room temperature. After staining, the cells were washed before imaging under microscope.

### Detection of reactive oxygen species

For the detection of the intracellular reactive oxygen species (ROS), cells (1 × 10^5^ cells/well) were cultured in triplicate using the 24-well plates. Following cell attachment, the medium was refreshed with NG (5.5 mM), NG + 16 (5.5 mM + 16ppm), HG (30 mM) or HG + 16 (30 mM + 16ppm) medium and further cultured for 24 h. ROS level was detected by the 2,7-dichlorofluorescein diacetate (DCFH-DA) fluorescent kit (Beyotime Institute of Biotechnology, China) [5]. Briefly, after adding 10 μM of DCFH-DA and incubation in the dark at 37°C for 20 min, cells were washed twice using the serum-free medium. The excitation at 488 nm and the emission at 525 nm were recorded by the microplate reader (Synergy HTX, Bio-tek Instruments Inc., Winooski, VT, USA). Finally, the ROS level was calculated as the ratio of OD488 to OD525.

### Quantitative real-time PCR assay

Cells (2 × 10^5^ cells/well) were cultured for 3 days in 6-well plates. The Trizol reagent (Invitrogen, USA) was employed for the extraction of total RNA. After reverse-transcription to cDNA as previously described [21], quantitative real-time PCR (qRT-PCR) amplification reaction was achieved on the Ex Taq DNA polymerase (TaKaRa, China). The primers used for the amplification were listed in Table 1. GAPDH was employed as the endogenous gene for normalization. The experiments were done in triplicate. The mRNA level was presented as 2^-ΔΔCt^ and normalized to the controls.

**Table 1:** The sequences of the primers for qPCR in the experiment

Gene	Forward primer	Reverse primer
ALP	5′ACCACCACGAGAGTGAACCA	5′CGTTGTCTGAGTACCAGTCCC
RUNX2	5′TGGTTACTGTCATGGCGGGTA	5′TCTCAGATCGTTGAACCTTGCTA
BMP2	5′TTCGGCCTGAAACAGAGACC	5′CCTGAGTGCCTGCGATACAG
COL1	5′GAGGGCCAAGACGAAGACATC	5′CAGATCACGTCATCGCACAAC
OCN	5′CACTCCTCGCCCTATTGGC	5′CCCTCCTGCTTGGACACAAAG
BMPR1A	5′CCTGGGCCTTGCTGTTAAATTCA	5′TCCACGATCCCTCCTGTGAT
BMPR1B	5′GTTGCTACTGGCTGTTTTGG	5′AAGTTCCCTGGGTGTCTG
BMPR2	5′GGCAGCAGTATACAGATAGGTG	5′CTGCCCTGTTACTGCCATTATT
SMAD1	5′GTATGAGCTTTGTGAAGGGC	5′TAAGAACTTTATCCAGCCACTGG
SMAD4	5′CTCCAGCTATCAGTCTGTCA	5′CCCGGTGTAAGTGAATTTCAAT
SMAD5	5′TCATCATGGCTTTCATCCCACC	5′GCTCCCCAACCCTTGACAAA
GAPDH	5′-TTCGACAGTCAGCCGCATCTT-3′	5′-ATCCGTTGACTCCGACCTTCA-3′

### Statistical analysis

All the data were expressed as mean ± standard deviation. The student’s t-test was used to evaluate the inter-group differences. The number of parallel samples is three in each cell assay. The difference was deemed statistically significant at a *P* values cutoff of < 0.05.

## Results

### The effect of Si ions on cell viability in HG-treated hBMSCs

**Figure 1:**
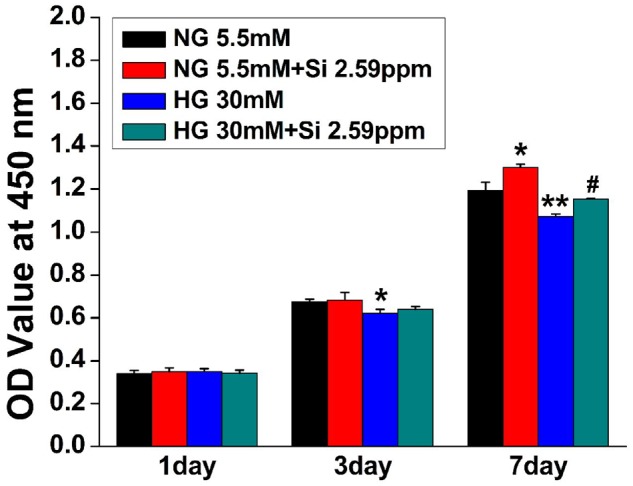
Effects of HG and Si on the proliferation of cultured hBMSCs. The data are represented as mean ± S.D. *P < 0.05 and **P < 0.01 compared with NG control group. #*P* < 0.05 compared with HG group. Si, silicon ion; NG, normal glucose; HG, high glucose; Si 2.59ppm represents the concentration of Si ions in medium

In order to determine whether Si ions affect the proliferation of hBMSCs under HG condition, Si ions were added into the HG medium with the final concentration of 2.59 ppm. Results depicted in Fig. 1 indicated that cell proliferation in NG + 2.59 was promoted compared with the cells in NG alone at day 7, while decreased proliferation of cells in HG was detected as compared with the cells in NG at day 3 and day 7. Notably, the proliferation of cells in HG + 2.59 did not show significant difference as compared with cells in NG alone and HG alone at day 1 and day 3, whereas significantly higher proliferation was observed at day 7 as compared with the cells in HG alone. The results confirmed that HG-inhibited proliferation of hBMSCs, and the treatment of 2.59 ppm Si ions indeed compensated the inhibition of cell proliferation caused by HG.

### The effect of Si ions on the expressions of osteogenic genes in HG-treated hBMSCs

**Figure 2:**
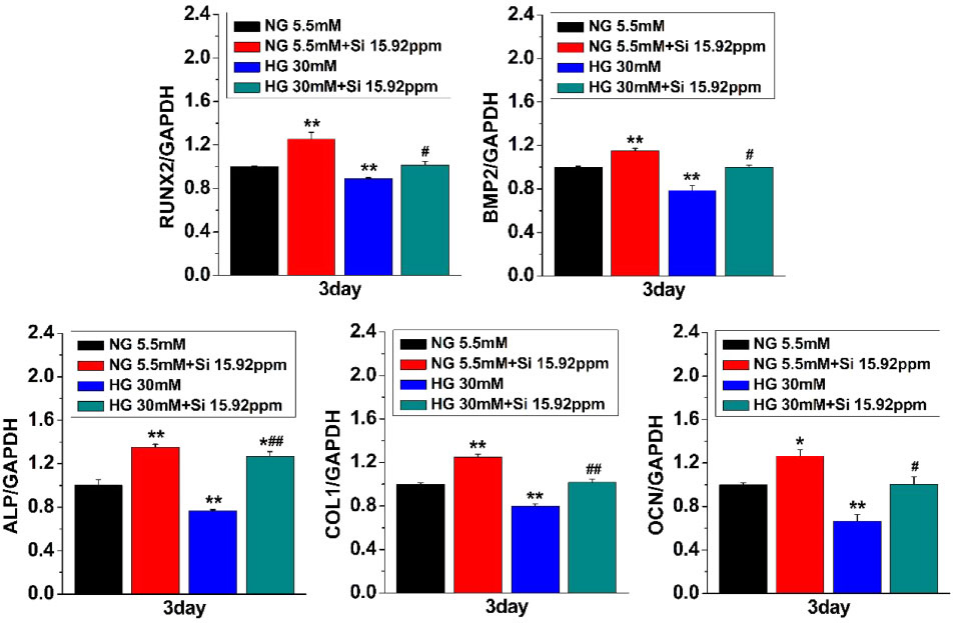
Effects of HG and Si on expressions of the osteogenic differentiation biomarkers in hBMSCs. Quantitative PCR was performed for expression of the osteogenic differentiation biomarkers, including *RUNX-2*, *BMP-2*, *ALP*, *COL-I* and *OCN* in cultured hBMSCs under different conditions. The data are represented as mean ± S.D. *P < 0.05 and **P < 0.01 compared with NG control group. #*P* < 0.05 and ##*P* < 0.01 compared with HG group. NG, normal glucose; HG, high glucose; Si 15.92ppm represents the concentration of Si ions in medium

To assess the impact of Si ions on the osteogenesis of hBMSCs under HG condition, cells were cultured for 3 days in different medium. As shown in Fig. 2, the bone-related genes of hBMSCs showed a higher expression levels in the group NG + 15.92 ppm than that in NG alone group, suggesting that 15.92 ppm concentration of Si ions stimulated osteogenic differentiation of hBMSCs. Nevertheless, the bone-related gene expression levels in HG-treated hBMSCs were significantly lower than that in NG alone-treated cells, implying that HG suppressed the osteogenic differentiation of hBMSCs. Furthermore, the expression of osteogenesis-related markers was markedly higher in the hBMSCs cultured in HG medium containing 15.92 ppm Si ions (HG + 15.92) than the HG alone. The results show that reduction of expressions of osteogenic genes under HG condition was compensated by the Si ions treatment.

### The effect of Si ions on ALP activity in HG-treated hBMSCs

**Figure 3:**
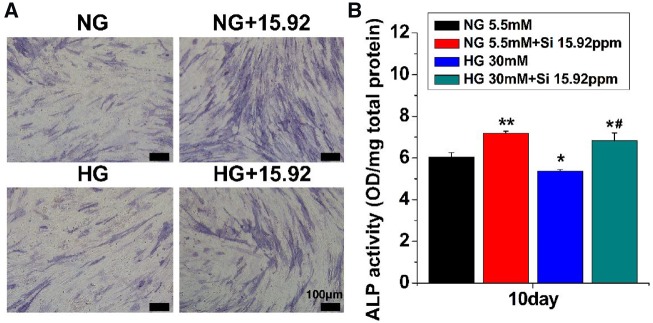
Effects of HG and Si on the ALP activity of hBMSCs. About 10 days after culture, staining (**A**) and quantitative analysis (**B**) of ALP were performed. The data are represented as mean ± S.D. *P < 0.05 and **P < 0.01 compared with NG control group. #*P* < 0.05 compared with HG group. Scale bar = 100μm. NG, normal glucose; HG, high glucose; Si 15.92ppm represents the concentration of Si ions in medium

The osteogenic differentiation of hBMSCs was evaluated in the presence of both HG and Si ions by performing the ALP activity assay and the ALP staining after seeding cells for 10 days. The results are shown in Fig. 3. Compared with NG alone, the ALP intensity was obviously higher in the cells cultured both in NG + 15.92 and in HG + 15.92 groups as compared with that in NG and HG groups (Fig. 3A). Quantitative ALP activity analysis showed similar results (Fig. 3B). The ALP activity of the cells cultured in HG was pointedly lower compared with cells cultured in NG alone. The ALP activity of cells in HG or NG with 15.92 ppm Si ions was higher compared with that in HG or NG alone, suggesting that suppression of ALP activity in HG-treated cells were compensated by Si ion treatment.

### The effect of Si ions on mineralization in HG-treated hBMSCs

**Figure 4:**
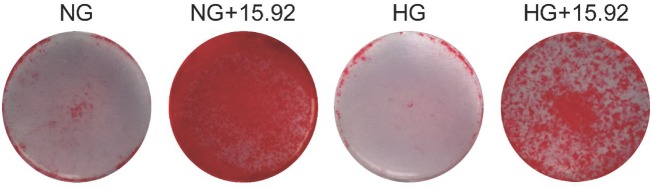
The formation of calcium nodules in cultured hBMSCs under different conditions. Calcium nodules were indicated by Alizarin red S staining in hBMSCs 21 days after culture. NG, normal glucose, 5.5mM; HG, high glucose, 30mM; 15.92 represents the concentration (15.92ppm) of Si ions in medium

Alizarin red S staining was used to evaluated newly formed nodules after 21 days. As shown in Fig. 4, the staining intensity was weaker in the cells cultured in NG or HG alone medium, whereas a strong staining was observed in the cells cultured in NG and HG with 15.92 ppm Si ions. In addition, as compared with NG alone or NG + 15.92, the staining intensity in HG or HG + 15.92 treated cells was obviously weaker, separately.

### Effects of Si ions on HG-mediated oxidative damage

**Figure 5:**
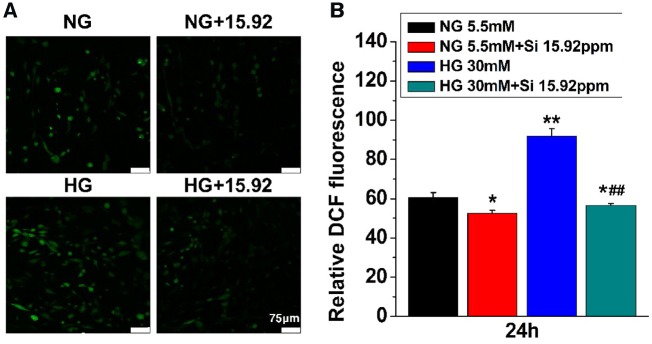
Effects of HG and Si on ROS level in hBMSCs. Staining (**A**) and quantitative measurement (**B**) of DCF fluorescence were performed in hBMSCs 24 hours after culture. The data are represented as mean ± S.D. *P < 0.05 and **P < 0.01 compared with NG control group. ##*P* < 0.01 compared with HG group; Scale bar = 75μm. NG, normal glucose; HG, high glucose; Si 15.92ppm represents the concentration of Si ions in medium

To confirm whether Si ions compensate the oxidative damage induced by HG in hBMSCs, ROS level was detected by using the 2,7-Dichlorofluorescein （DCF） fluorescence staining. As shown in Fig. 5A, the level of the intracellular ROS was upgraded in HG group as compared with NG group. However, the intensity of ROS was clearly reduced in both 15.92 ppm Si ions treated cells as compared with that in HG or NG alone cultured cells.

The quantitative measurement of DCF fluorescence was shown in Fig. 5B. The DCF values of hBMSCs were significantly higher in HG than NG indicating HG-induced oxidative damage in hBMSCs. However, the intensity of ROS was obviously reduced by the treatment of 15.92 Si ions as compared with NG or HG alone, suggesting that HG-induced oxidative damage in hBMSCs was alleviated by Si ions treatment.

### BMP2 signaling pathway-related gene expression

To explore the regulatory role of BMP2 signaling in osteogenic differentiation induced by Si ions, hBMSCs were treated with 15.92 ppm Si ions in the presence of BMP2 signaling inhibitor noggin and under NG or HG condition for 3 days. The relative mRNA expression levels of BMP2 signaling pathway-related genes including BMP2 signaling receptors (*BMPR1A*, *BMPR1B* and *BMPR2*) and its downstream genes *SMAD1*, *SMAD4* and *SMAD5* were quantitated using qRT-PCR assay.

**Figure 6:**
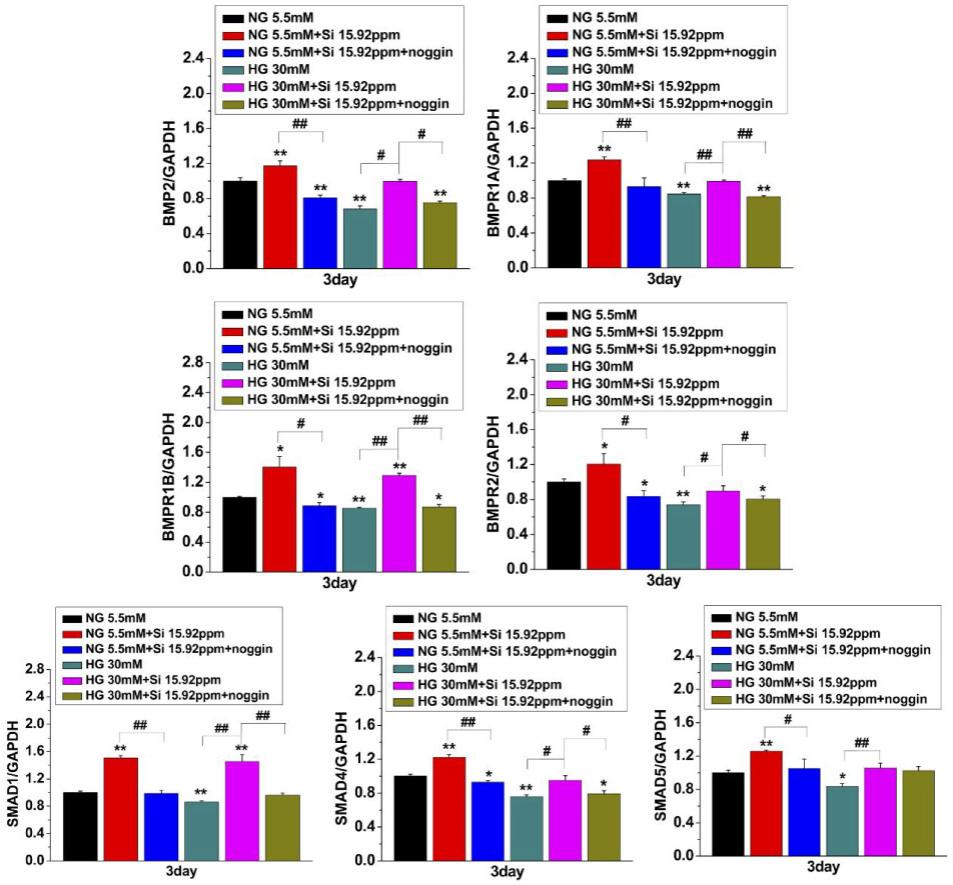
Effects of HG and Si on expression of BMP2-related signaling pathway genes in hBMSCs. hBMSCs were treated with BMP2 signaling inhibitor noggin and Si ions (15.92 ppm) under NG or HG condition for 3 days. qPCR was performed for expression of BMP2 signaling pathway genes *BMPR1A*, *BMPR1B*, *BMPR2*, *SMAD1*, *SMAD4* and *SMAD5*. The data are represented as mean ± S.D. *P < 0.05 and **P < 0.01 compared with NG control group. #*P* < 0.05 and ##*P* < 0.01 compared with HG group. NG, normal glucose; HG, high glucose; Si 15.92ppm represents the concentration of Si ions in medium

**Figure 7:**
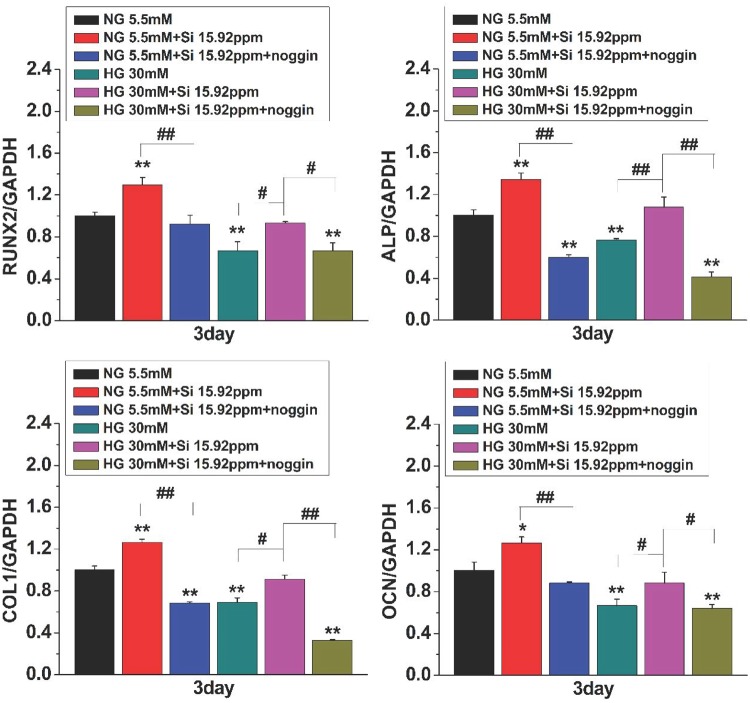
Effects of noggin on osteogenic marker genes in HG- and Si-treated hBMSCs. hBMSCs were treated with BMP2 signaling inhibitor noggin and Si ion (15.92ppm) under NG or HG condition for 3 days. qPCR was performed for expressions of osteogenic marker genes *RUNX2*, *ALP*, *COL1* and *OCN* in hBMSCs. The data are represented as mean ± S.D. *P < 0.05, **P < 0.01, #*P* < 0.05, ##*P* < 0.01. NG, normal glucose; HG, high glucose; Si 15.92ppm represents the concentration of Si ions in medium

The results in Fig. 6 indicated that the mRNA levels of all target genes were noticeably upregulated by the treatment of 15.92 ppm Si ions as compared with NG or HG alone. More interestingly, Si ions induced increase of the expression of BMP2 signaling pathway genes was significantly attenuated by addition of noggin, suggesting that the activity of Si ions in promoting osteogenesis and compensating the HG effect on hBMSCs is closely related to activating BMP2 signaling pathway by stimulating some BMP receptors expression. Moreover, as shown in Fig. 7, the relative mRNA expression levels of BMP2 signaling pathway downstream genes such as *RUNX2*, *ALP*, *COL1* and *OCN* were also decreased by noggin treatment in NG + 15.92 and HG + 15.92 groups. All the results indicate an association between BMP2 signaling and Si ion-induced osteogenic differentiation in hBMSCs cultured under HG condition.

### Ionic concentration

The concentrations of Ca and Si ions in media are shown in Table 2. We can see the concentration of Ca ions in different medium have no significant difference, on the contrary, the Si ions significantly higher than that in the NG or HG medium. Thus, the effect of Ca ions was not our concern in the current work.

**Table 2:** Ions concentrations of the different medium (μg/ml)

	NG	NG + 2.59	NG + 15.92	HG	HG + 2.59	HG + 15.92
Ca	70.01	70.14	70.79	70.01	70.14	70.79
Si	0	2.59^a^	15.92^a^	0	2.59^a^	15.92^a^

NG: normal glucose; HG: high glucose; 2.59 and 15.92 represent the concentration of Si ions.

^a^Indicates that the Si ion concentration in medium is significantly higher than that in NG or HG medium (*P*  <  0.01).

## Discussion

Studies have shown that diabetes can affect bone metabolism through multiple pathways, and hyperglycemia has been demonstrated to cause delayed bone healing [3, 22–24]. hBMSCs governs multiple functions such as the self-renewal, hematopoiesis support, osteogenesis and immunity [25, 26]. Despite their application as seed cells for tissue engineering, early reports conveyed that HG could suppress the growth and osteogenic differentiation of hBMSCs [3, 4]. Moreover, BMSCs cultured in HG medium showed significant decrease of BMP2 gene expression, in which the activation of BMP signaling pathways was inhibited [6]. Biomaterials play a key role in the regulation of cell proliferation and differentiation of stem cells. In our previous work, we found that Si ions released from silicate bioceramics induce the cell growth and osteogenic differentiation of hBMSCs [9–12, 15]. However, the effect of Si ions on the growth and osteogenesis of hBMSCs under HG condition has not been reported. Therefore, we assumed that the Si ions from silicate bioceramics might compensate the HG-induced inhibition of the proliferation and osteogenic differentiation in hBMSCs. In the present study, we successfully confirmed that Si ions indeed have the activity to regulate osteogenic differentiation of hBMSCs not only under NG condition but also under HG condition, and inhibited the negative effect of HG through modulation of BMP2 signaling pathway.

BMP2 is a vital osteogenic marker and is generally expressed at early stage of osteogenic differentiation [27]. BMP2 and its receptor signaling pathway play a key role in the osteogenic differentiation [28, 29]. Previous study reported that BMP2 could affect the expression of Runx2 and Osterix [30]. BMP-SMAD signal pathway can regulate the whole process of osteoblast formation [31]. *In vitro* and animal experiments have demonstrated that inhibiting the BMP-SMAD pathway can significantly down-regulate the expression of Osterix and Runx2 [32, 33]. In addition, Runx2 interacts with SMAD1 and SMAD5 to enhance the expression of osteogenic genes [33]. Herein, we demonstrated that Si ions markedly stimulate the expression of Runx2, BMP2 receptors and SMADs, and the increased expression of these osteogenic markers was significantly reduced by noggin (Fig. 7), which suggests that the osteogenic differentiation of hBMSCs might be regulated via activation of BMP2 signaling pathway. The initiation of the canonical BMP signaling which play a significant role in the activation of target cells by BMPs is triggered by the association of BMP receptors (type I and type II) with BMPs followed by subsequent activation of the BMP-SMAD signaling [34, 35]. Thus, we supposed that the Si ions activated BMP2 signaling pathway possibly by stimulating the expression of BMP2 and BMP2 receptors. We found that the Si ions treatment increased the expression of the downstream genes of BMP2 signaling pathway such as SMAD1, SMAD4 and SMAD5 (Fig. 6). The complexes obtained by the association of SMAD1 and SMAD5 with SMAD4, are translocated into the nucleus where they act as transcription activators of target genes [34, 35], such as Runx2 responsible for bone formation and other genes associated with osteogenic differentiation ALP, COL1 and OCN [36]. These data further confirmed that Si ions stimulated the osteogenic differentiation of hBMSCs under HG condition via activation of BMP2 signaling pathway.

Oxidative stress is induced by elevated level of ROS that interrupts the reduction–oxidation (redox) balance in the cells [37]. Excessive production of ROS damages cell, and ROS is known to exert different functions in aging and its related complications [38]. Previous studies indicated that ROS production play a relevant role in mineral tissue homeostasis and is involved in bone remodeling via induction of bone resorption [39–44]. The data from *in vitro* studies showed that HG could increase the level of ROS in hBMSCs [4, 5]. Therefore, inhibition of ROS production may be one of the key steps for reducing cell damage induced by HG environment. Interestingly, our findings indicated that the treatment with Si ions pointedly lessened the production of ROS in hBMSCs (Fig. 5), which is the first report on the inhibition of ROS production by ions released from bioceramics.

## Conclusion

In this work, we established that the inhibition of the proliferation and osteogenic differentiation of hBMSCs by HG environment was partially compensated by the treatment of Si ions released from CS bioceramics. Two possible mechanisms of the Si ions on the compensation of HG effect were proposed. On one side, Si ions stimulated osteogenic differentiation of hBMSCs under HG condition through activation of BMP2 signaling pathway. On the other side, Si ions inhibited ROS production of hBMSCs induced by HG treatment. Our findings suggested that the release of bioactive Si ions from silicate-based biomaterials reduces the cell damage caused by the HG environment, therefore, the silicate bioceramics might be used as hBMSCs carriers for bone tissue engineering applications in diabetic patients.

## Funding

This work was supported by the Natural Science Foundation of China [Grant No. 31271054 and 31770980].

*Conflict of interest statement*. None declared.
